# Virtual supersampling as post-processing step preserves the trabecular bone morphometry in human peripheral quantitative computed tomography scans

**DOI:** 10.1371/journal.pone.0212280

**Published:** 2019-02-13

**Authors:** Friederike A. Schulte, Patrik Christen, Sandro D. Badilatti, Ian Parkinson, Sundeep Khosla, Jörg Goldhahn, Ralph Müller

**Affiliations:** 1 Institute for Biomechanics, ETH Zurich, Zurich, Switzerland; 2 SA Pathology and University of Adelaide, Adelaide, Australia; 3 Division of Endocrinology, Diabetes, Metabolism and Nutrition, Department of Internal Medicine, Mayo Clinic, Rochester, MN, United States of America; University of Notre Dame, UNITED STATES

## Abstract

In the clinical field of diagnosis and monitoring of bone diseases, high-resolution peripheral quantitative computed tomography (HR-pQCT) is an important imaging modality. It provides a resolution where quantitative bone morphometry can be extracted *in vivo* on patients. It is known that HR-pQCT provides slight differences in morphometric indices compared to the current standard approach micro-computed tomography (micro-CT). The most obvious reason for this is the restriction of the radiation dose and with this a lower image resolution. With advances in micro-CT evaluation techniques such as patient-specific remodeling simulations or dynamic bone morphometry, a higher image resolution would potentially also allow the application of such novel evaluation techniques to clinical HR-pQCT measurements. Virtual supersampling as post-processing step was considered to increase the image resolution of HR-pQCT scans. The hypothesis was that this technique preserves the structural bone morphometry. Supersampling from 82 μm to virtual 41 μm by trilinear interpolation of the grayscale values of 42 human cadaveric forearms resulted in strong correlations of structural parameters (R^2^: 0.96–1.00). BV/TV was slightly overestimated (4.3%, R^2^: 1.00) compared to the HR-pQCT resolution. Tb.N was overestimated (7.47%; R^2^: 0.99) and Tb.Th was slightly underestimated (-4.20%; R^2^: 0.98). The technique was reproducible with PE_%CV_ between 1.96% (SMI) and 7.88% (Conn.D). In a clinical setting with 205 human forearms with or without fracture measured at 82 μm resolution HR-pQCT, the technique was sensitive to changes between groups in all parameters (p < 0.05) except trabecular thickness. In conclusion, we demonstrated that supersampling preserves the bone morphometry from HR-pQCT scans and is reproducible and sensitive to changes between groups. Supersampling can be used to investigate on the resolution dependency of HR-pQCT images and gain more insight into this imaging modality.

## Introduction

Quantitative assessment of the trabecular bone microstructure is a valuable tool in bone research because a number of bone diseases act directly on the trabecular bone surface which causes alterations in the bone microstructure.

The current standard technology to quantify human three-dimensional bone morphology is micro-computed tomography (micro-CT) *ex vivo* where no radiation issues have to be respected. With advances in *in vivo* imaging technologies, the quantitative determination of the bone morphology has entered the clinical setting. High-resolution, peripheral quantitative computed tomography (HR-pQCT) is a promising clinical tool for the monitoring of the microstructure in bone diseases and their treatments *in vivo* in patients. HR-pQCT provides an image resolution where single trabeculae can be resolved and the bone microstructure can be quantified using the techniques originally developed for micro-CT [1,2]. Trabecular and cortical microstructural and biomechanical parameters gained from HR-pQCT scans have been validated in comparison to scans obtained using micro-CT at distal radius, tibia and calcaneus [1,3–7]. Metcalf et al. [3] found, comparing trabecular structural parameters of HR-pQCT with micro-CT, very strong correlations of BV/TV, and moderate correlations for Tb.Th and Tb.N. Christen et al. [4] developed a biomechanical analysis tool for micro-CT resolutions [5] which provides detailed insight into the reasons for a certain form of the bone microstructure. Assuming bone forms tissue at high-load locations and resorbs tissue at low-load locations, the approach finds a set of load cases that lead to the most uniform tissue loading using optimization [6–8]. Christen et al. investigated the tool’s voxel size dependency, reproducibility, and sensitivity on HR-pQCT data and showed with this that their tool can be applied at HR-pQCT resolutions.

Further image processing techniques that were developed for micro-CT images at nominal image resolutions of about 10 μm require voxel sizes smaller than provided by HR-pQCT. This concerns the assessment of three-dimensional bone formation and resorption rates that has been validated for *in vivo* micro-CT [9]. With the availability of time-lapsed scans of a patient, it would be great to have access to his dynamic local bone changes per week or month. However, assuming human mineral apposition rates of about 10 μm per week [10], it would take 8 weeks of continuous bone growth until a full new voxel (82 μm) was filled with bone mass. Only dividing the voxel size by two would generate a huge benefit in reducing the error caused by partial volumes effects.

Also, having access to *in silico* simulation tools that use a patient’s microstructure as input and predict how the bone evolves over time under a certain treatment can be of great use for both physician and patient. Such subject-specific *in silico* simulations to predict and possibly adapt a subject’s treatment regime have been explored on preclinical *in vivo* micro-CT datasets at resolutions of about 10 μm [11].

A number of studies have used downscaling to investigate on the resolution dependency of the bone morphometry [12–16]. Little attention has been paid to the reverse effect, i.e. the upscaling of reconstructed images. Such upscaling could provide a technique so that pQCT scans get access to tools currently not applicable at clinical image resolutions. Further, the investigation of such analysis can help gain more insight into the direct effects of the image resolution on bone morphometry. Here we propose a technique to supersample HR-pQCT images. We hypothesize that the structural bone morphometry is preserved by the proposed technique and we investigate the effect on bone morphometry in terms of precision and sensitivity.

## Materials and methods

### Precision

To test for the precision of bone morphometry parameters on supersampled images, a study consisting of repeated HR-pQCT scans of 14 human radii was re-used [17]. In short, the 14 formalin-fixed intact human cadaveric forearms had been imaged each 3 times with repositioning after each measurement with a HR-pQCT scanner (XtremeCT, Scanco Medical AG, Brüttisellen, Switzerland) providing an isotropic nominal resolution of 82 μm.

**Fig 1:**
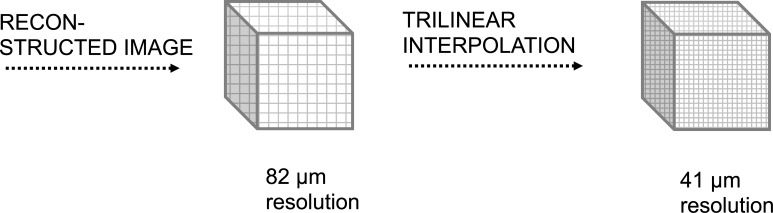
Schematic illustration of the technical proceeding.

All raw datasets underwent a 3D Laplace-Hamming filter (Hamming cut-off frequency = 0.4, weighting factor = 0.5) [17]. The repeated scans were registered to allow evaluation of the same regions of interest. The registered images were supersampled by a factor of 2 (using trilinear interpolation), resulting in a virtual 41 μm image resolution (Fig 1). Both the original (82 μm) and the supersampled (41 μm) grayscale images were segmented at a global threshold of 400/1000 of the maximal gray scale value.

The trabecular compartment was separated from the cortex using a distance-based segmentation algorithm [18]. Static morphometry parameters bone volume fraction (BV/TV), specific bone surface (BS/BV), trabecular thickness (Tb.Th), trabecular spacing (Tb.Sp), trabecular number (Tb.N), structural model index (SMI), degree of anisotropy (DA) and connectivity-density (Conn.D) were calculated in both the measured 82 μm and the supersampled 41 μm resolution images.

The reproducibility of bone morphometric indices at both image resolutions was expressed in precision errors PE_SD_, PE_%CV_, the intraclass correlation coefficient ICC and the 95%-confidence intervals ICC_lower_ and ICC_upper_ [19]. Furthermore, the bone morphometry of images at virtual 41 μm was compared to the original image resolution of 82 μm. Significant differences to the measured image resolution of 82 μm were calculated by paired Students t-test. Furthermore, linear regression analysis was performed between bone morphometry from the 82 μm and 41 μm image resolutions. All statistical comparisons were performed using R (Statistical Software package, Vienna, Austria [20]). P-values less than 0.05 were considered significant.

### Sensitivity

Sensitivity of the proposed method was assessed by reanalysis of a prior study, where 100 postmenopausal women experienced a Colles’ fracture at median age 63 years [21]. The fracture cases were frequency-matched to 105 age-matched control women at median age 62 years. Written informed consent was obtained from all subjects before participation in the study. The study was approved by the Mayo Clinics Institutional Review Board and the present reanalysis was based on de-identified data. The distal radius of the study subjects was measured by HR-pQCT at an isotropic voxel size of 82 μm (XtremeCT, Scanco Medical AG, Brüttisellen, Switzerland). In all study subjects, the non-fractured side (fractured subjects) or the non-dominant arm (controls) was assessed.

All raw datasets underwent a 3D Laplace-Hamming filter (Hamming cut-off frequency = 0.4, weighting factor = 0.5) [17]. Afterwards, the images were upscaled (supersampled) by a factor of 2 using trilinear interpolation. Both the original and the interpolated grayscale images were segmented at a global threshold of 400/1000 of the maximal gray scale value [21]. A trabecular mask was used to extract morphometric parameters in the trabecular compartment. The structural parameters BV/TV, BS/BV, Tb.Th, Tb.Sp, Tb.N, SMI, DA and Conn.D were calculated. Unpaired Student’s t-test was used to detect differences in means between fracture cases and controls. Linear correlations between 82 μm and 41 μm resolutions were assessed. Pairwise comparisons between resolutions were calculated.

## Results

### Precision

All precision values including mean and standard deviation, PE_SD_, PE_%CV_ with its upper and lower confidence intervals as well as the ICC values including their upper and lower confidence intervals can be found in Table 1. At 82 μm resolution, precision errors of repeated human *ex vivo* forearm scans ranged from 2.05% (SMI) to 8.12% (Conn.D). The ICCs ranged between 0.867 (Tb.Th) and 0.995 (BV/TV).

**Table 1:** Reproducibility of static bone morphometric indices, expressed as precision error of the standard deviation (PE_SD_), coefficient of variation (PE_%CV_) and intra-class correlations (ICC). The upper part of the table shows the reproducibility at original image resolution (82 μm) and the lower part of the table the reproducibility at virtual 41 μm resolution.

Parameter	Mean ± SD	PE_SD_	PE_%CV_	PE_lower_	PE_upper_	ICC	ICC_lower_	ICC_upper_
Original image resolution (82 μm)								
**BV/TV [%]**	17.67 ± 5.91	0.43	2.99	2.377	4.050	0.995	0.988	0.998
**BS/BV [mm**^**2**^**/mm**^**3**^**]**	13.77 **±** 1.77	0.41	2.71	2.154	3.671	0.947	0.877	0.981
**Tb.Th [mm]**	0.22 **±** 0.02	0.01	3.71	2.942	5.014	0.867	0.712	0.951
**Tb.Sp [mm]**	0.84 **±** 0.38	0.05	3.79	3.011	5.131	0.984	0.960	0.994
**Tb.N [1/mm]**	1.26 **±** 0.28	0.04	3.75	2.974	5.069	0.986	0.965	0.995
**SMI**	1.87 **±** 0.37	0.04	2.05	1.625	2.770	0.989	0.974	0.996
**DA**	1.35 **±** 0.08	0.03	2.06	1.636	2.787	0.880	0.736	0.956
**Conn.D**	2.24 **±** 0.79	0.17	8.12	6.443	10.980	0.985	0.964	0.995
Virtual high resolution (41 μm)								
**BV/TV [%]**	18.411**±** 6.1618	0.468	3.197	2.537	4.324	0.995	0.989	0.998
**BS/BV [mm**^**2**^**/mm**^**3**^**]**	13.494 **±** 1.7371	0.415	2.737	2.172	3.702	0.945	0.871	0.980
**Tb.Th [mm]**	0.233 **±** 0.0182	0.008	3.818	3.030	5.163	0.817	0.619	0.931
**Tb.Sp [mm]**	0.824 **±** 0.3284	0.046	3.147	2.497	4.256	0.983	0.959	0.994
**Tb.N [1/mm]**	1.154 **±** 0.2232	0.029	3.172	2.517	4.289	0.991	0.977	0.997
**SMI**	1.970 **±** 0.4235	0.038	1.958	1.554	2.648	0.992	0.980	0.997
**DA**	1.367 **±** 0.0814	0.033	2.204	1.749	2.981	0.872	0.720	0.953
**Conn.D**	1.983 **±** 0.7581	0.150	7.882	6.255	10.660	0.983	0.960	0.994

**Fig 2:**
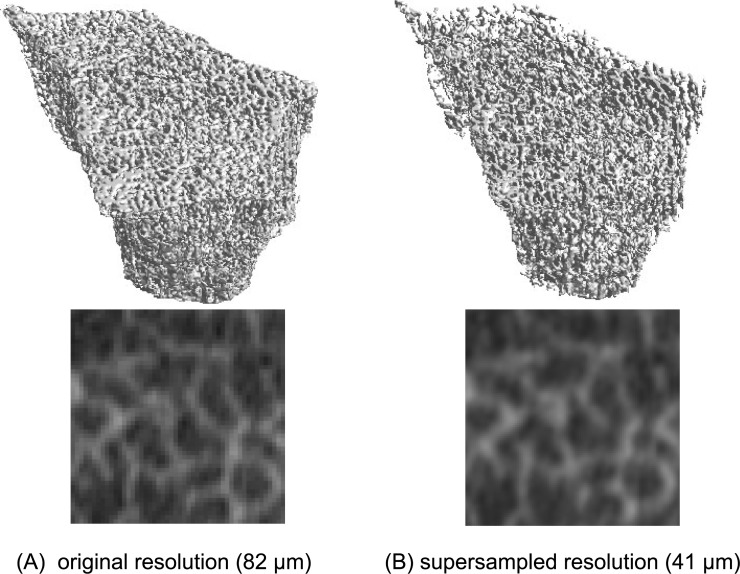
(A) Human forearm measured *ex vivo* at 82 μm resolution in 3D (upper) and 2D (lower) (B) same virtually reconstructed human forearm at 41 μm resolution.

**Fig 3:**
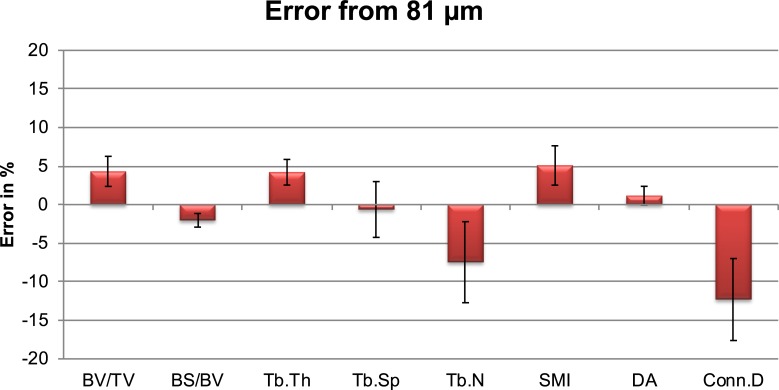
Absolute mean errors in % where the original resolution serves as baseline.

Supersampling (41 μm) led to precision errors between 1.96% (SMI) and 7.88% (Conn.D), with ICCs between 0.817 (Tb.Th) and 0.995 (BV/TV). Fig 2 shows a representative forearm structure at (A) 82 μm and at (B) 41 μm resolution. On the left, at 82 μm resolution, structures present a more pixelated structure. Trabeculae seem thicker and prone to disconnections. On the right, the virtually reconstructed image resembles more a micro-CT image with smooth structures. Fig 3 represents the mean errors of structural indices at virtual 41 μm resolution from the original indices at 82 μm resolution. Supersampled images lead to a higher BV/TV (4.31%, R^2^: 1.00), higher Tb.Th (4.20%, R^2^: 0.98), lower Tb.N (-7.47%, R^2^: 0.99) and a lower Conn.D (-12.31%, R^2^: 0.98). Table 2 lists the coefficients of determination R^2^, the absolute %-errors between 41 μm and 82 μm resolution and the intercept functions by which the 41 μm resolution parameters have to be scaled to fit the original 82 μm voxel size parameters.

**Table 2:** Coefficients of determination R^2^, intercept functions and absolute errors (mean ± standard deviation) between 82 μm and 41 μm resolution (*p < 0.05, **p < 0.01, ***p < 0.001).

Parameter	R^2^	intercept function	absolute error in % (p-value)
**BV/TV [%]**	1.00	1.00x + 0.0041	4.31 **±** 1.95 (***)
**BS/BV [mm**^**2**^**/mm**^**3**^**]**	0.99	0.98x + 0.013	-2.03 **±** 0.88 (***)
**Tb.Th [mm]**	0.98	0.91x+0.03	4.20 **±** 1.66 (***)
**Tb.Sp [mm]**	0.99	0.87x + 0.093	-0.64 **±** 3.62 (-)
**Tb.N [1/mm]**	0.99	0.79x+0.16	-7.47 **±** 5.26 (***)
**SMI**	0.99	1.10x – 0.14	5.08 **±** 2.55 (***)
**DA**	0.96	1.00x +0.017	1.18 **±** 1.20 (***)
**Conn.D**	0.98	0.95x - 0.15	-12.31 **±** 5.32 (***)

### Sensitivity

**Fig 4:**
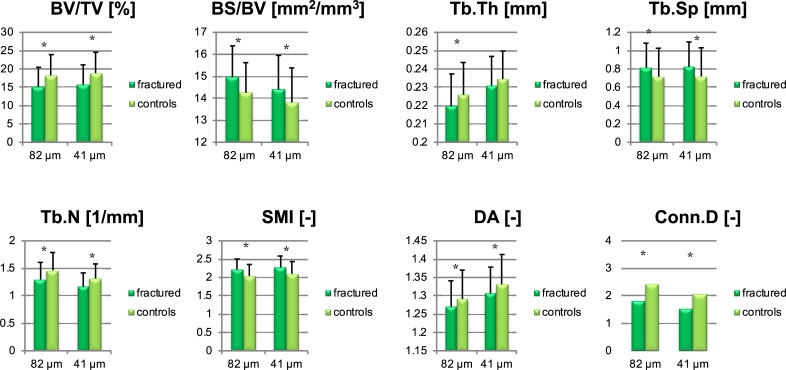
Means with standard deviation between controls and fractured cases at original 82 μm resolution and supersampled 41 μm resolution (*p < 0.05).

Table 3 and Fig 4 show the results of the clinical data (fractured /non-fractured) measured at 82 μm image resolution and supersampled at virtual 41 μm resolution. At 82 μm resolution, bone morphometry in the fractured group performed in all morphometric parameters significantly worse than in the control group, i.e. lower BV/TV, Tb.Th, Tb.N and higher BS/BV, Tb.Sp and Conn.D (p < 0.05). Tb.Th differed only 2.71% between groups but was still significant.

**Table 3:** Sensitivity. Comparison between 100 postmenopausal women and 105 controls in terms of their trabecular bone structure parameters; values are given as mean **±** standard deviation and mean %-difference between groups (*p < 0.05, **p < 0.01, ***p < 0.001).

Parameter	fracture cases (mean ± standard deviation)	control cases (mean ± standard deviation)	mean %-difference (p-value)
Original image resolution (82 μm)			
**BV/TV [%]**	15.15±5.31	18.28±5.66	-17.16***
**BS/BV [mm**^**2**^**/mm**^**3**^**]**	14.79±1.56	14.25±1.56	5.06**
**Tb.Th [mm]**	0.22±0.02	0.23±0.02	-2.71*
**Tb.Sp [mm]**	0.81±0.27	0.72±0.31	13.26*
**Tb.N**	1.30±0.31	1.46±0.33	-11.32***
**SMI**	2.22±0.28	2.05±0.31	8.65***
**DA**	1.27±0.07	1.29±0.08	-1.63*
**Conn.D**	1.82±1.01	2.44±1.05	-25.39***
Supersampled resolution (41 μm)			
**BV/TV [%]**	15.75±5.38	18.90±5.66	-16.63***
**BS/BV [mm**^**2**^**/mm**^**3**^**]**	14.39±1.41	13.82±1.37	4.13**
**Tb.Th [mm]**	0.23±0.02	0.23±0.02	-1.48
**Tb.Sp [mm]**	0.83±0.27	0.72±0.31	14.83**
**Tb.N**	1.17±0.25	1.31±0.27	-11.23***
**SMI**	2.28±0.31	2.10±0.33	8.42***
**DA**	1.31±0.07	1.33±0.08	-1.84*
**Conn.D**	1.53±0.85	2.04±0.89	-25.01***

At 41 μm resolution, the same structural parameters were assessed. In the between-groups-comparison (fractured vs. non-fractured), the same significances as in the 81 μm comparison were detected. Only the difference of Tb.Th was with 1.48% not significant any more.

**Fig 5:**
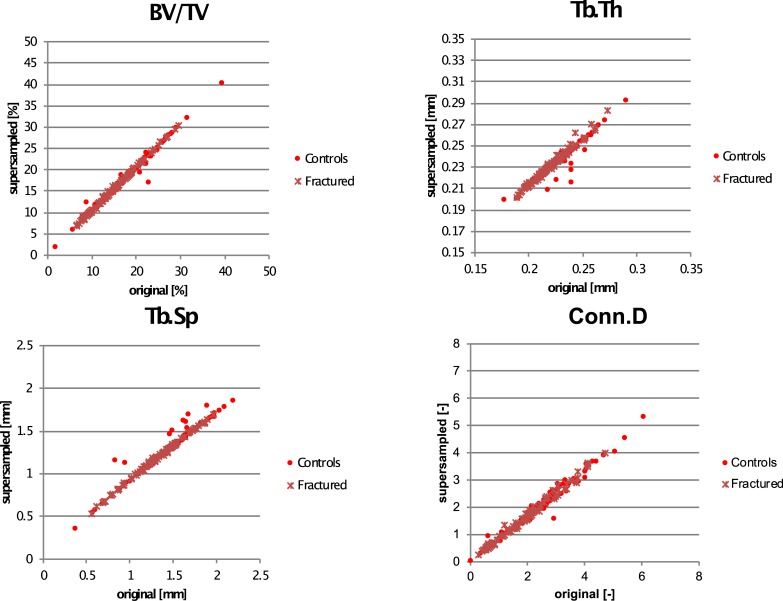
Correlations between original 82 μm resolution and supersampled 41 μm resolution of BV/TV, Tb.Th, Tb.Sp and Conn.D.

Linear regression analysis between the original 82 μm and supersampled 41 μm resolution images revealed R^2^-values between 0.94 and 0.99 (Table 4 and Fig 5). At 41 μm resolution, BV/TV was overestimated at 4.3% (R^2^: 0.99). Specific bone surface (BS/BV) was 3.3% lower than at 82 μm resolution (R^2^: 0.96). Tb.Th was overestimated with 4.56% (R^2^: 0.94) and Tb.N was underestimated with 9.39% (R^2^: 0.97). All pairwise comparisons between resolutions were significant (p<0.05). The intercept functions by which the supersampled parameters have to be scaled can be found in Table 4.

**Table 4:** Sensitivity. Linear regression analysis between 82 μm and 41 μm resolution is shown as coefficient of determination R^2^, the intercept functions of the structural parameters and mean %-errors (*p < 0.05, **p < 0.01, ***p < 0.001).

Parameter	R^2^	intercept function	absolute error in % (p-value)
**BV/TV [%]**	0.99	1x + 0.6	4.30***
**BS/BV [mm**^**2**^**/mm**^**3**^**]**	0.96	0.88x + 1.3	-3.30***
**Tb.Th [mm]**	0.94	0.85x + 0.043	4.56***
**Tb.Sp [mm]**	0.98	0.99x + 0.017	1.15*
**Tb.N [1/mm]**	0.97	0.8x + 0.13	-9.39***
**SMI**	0.97	1.1x – 0.064	2.59***
**DA**	0.99	1x + 0.003	2.96***
**Conn.D**	0.98	0.83x + 0.017	-1.70***

## Discussion

In this study, we showed that linear supersampling of HR-pQCT scans at 82 μm voxel size to virtual 41 μm voxel size as a post-processing step is reproducible and sensitive to changes between groups. Human radius trabecular BV/TV and other trabecular parameters assessed at an isotropic 82 μm resolution were used as input structures. They were supersampled at virtual 41 μm resolution by trilinear interpolation.

82 μm HR-pQCT BV/TV and supersampled BV/TV had a strong correlation (R^2^ = 0.99–1.00) and an absolute error of 4.30% - 4.31%. BV/TV at 41 μm voxel size was consistently higher than at 82 μm voxel size. This phenomenon supports the findings of previous studies [3,13,22,23] which validated HR-pQCT BV/TV with ‘gold standard’ micro-CT at distal radius, tibia and calcaneus. Metcalf et al. [3] suggested that the lower BV/TV at HR-pQCT resolution results either from the global threshold, beam hardening artifacts or the lower signal-to-noise-ratio resulting from a higher number of partial volume voxels. In our study, we can exclude both beam hardening artifacts and threshold-related differences. Thus we can specify the reason for the lower BV/TV in HR-pQCT images to the lower signal-to-noise-ratio where those parts of the bone volume that do not fill up a whole voxel get lost.

In our study, Tb.Th was consistently overestimated (4.2% - 4.56%) at virtual 41 μm resolution and Tb.N was consistently underestimated (-7.47%—-9.3%). Again, these findings fit with previous studies which found that HR-pQCT obtained Tb.Th is lower and Tb.N is higher than micro-CT obtained parameters [3,24]. As our study scaled from lower to higher image resolution, again we can point the reason for those findings to the lower sampling frequency resulting where the partial volumes take more effect.

Conn.D was not affected by the sensitivity study but increased at 41 μm resolution at the precision study. A reason for this can be that fine structures connected with each other can be grasped better at a higher sampling frequency which is obtained by supersampling of the image resolution.

Precision errors of HR-pQCT scanned volumes using registration have been reported between 3% and 6% [17,25–28]. Our findings are consistent with the reportings in the literature (PE_%CV_: 2.05–3.79; Conn.D: 8.12). Supersampled 41 μm voxel sizes resulted in slightly improved precision errors (PE_%CV_: 1.96–3.81; Conn.D: 7.88). With such small differences, we conclude that the sampling frequency itself seems to play a minor role in the reproducibility of HR-pQCT scans.

We recognize there are several limitations to the study. First, we validated the virtual 41 μm resolution by comparison to the original 82 μm HR-pQCT input. A comparison to ‘gold standard’ micro-CT scans would potentially have given even more insight into possible benefits in terms of accuracy of the proposed technique. However, several studies have investigated on the differences between micro-CT and HR-pQCT and found strong correlations [3,13,23,29,30]. Nevertheless, with this constraint, our conclusions can only be drawn with respect to HR-pQCT image quality. Second, we used the first generation Xtreme-CT device which has an isotropic voxel size of 82 μm [31] while the second generation Xtreme-CT would have an isotropic resolution of 61 μm itself [32]. Third, our analysis was restricted to structural parameters and further evaluation is needed to investigate whether such supersampling can also be applied for mechanical, dynamic or other structural parameters than those presented.

In conclusion, we have investigated the precision and sensitivity of a supersampling method for human HR-pQCT scans at 82 μm voxel sizes. The investigation showed minimal differences between image resolutions of the static bone morphometry which can be assigned to the sampling frequency. The advantage of this technique is that other disruptive factors than the sampling frequency can be denied. In terms of sensitivity, all structural parameters except trabecular thickness revealed the same differences between patient groups as at original HR-pQCT resolution. We conclude that the proposed supersampling technique is reproducible and sensitive to changes between groups.

## Supporting information

S1 File. Data All relevant data including statistical analyses and R scripts. (ZIP)
