# Supplementary figures and images for: Virtual supersampling as post-processing step preserves the trabecular bone morphometry in human peripheral quantitative computed tomography scans
Source: PLoS One. 2019 Feb 13;14(2):e0212280. doi: 10.1371/journal.pone.0212280 (PMC6373954; doi:10.1371/journal.pone.0212280)

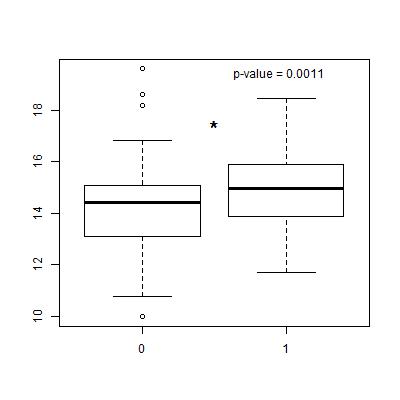

Supplement: S1 File. Data — (ZIP) [file pone.0212280.s001.zip › Evaluation/UpSampleMayo/doc/stat/img/BS.BV_.jpg]

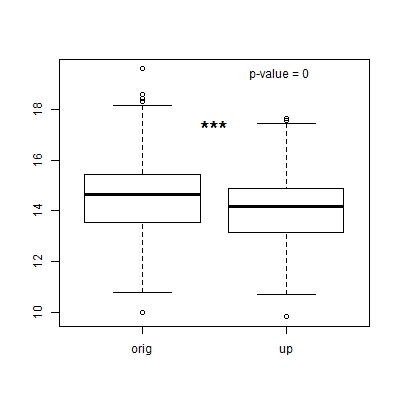

Supplement: S1 File. Data — (ZIP) [file pone.0212280.s001.zip › Evaluation/UpSampleMayo/doc/stat/img/BSBVpairwise.jpg]

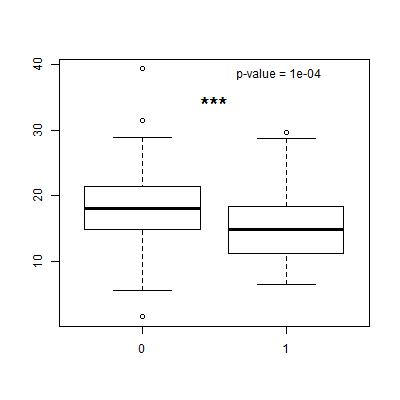

Supplement: S1 File. Data — (ZIP) [file pone.0212280.s001.zip › Evaluation/UpSampleMayo/doc/stat/img/BV.TV_.jpg]

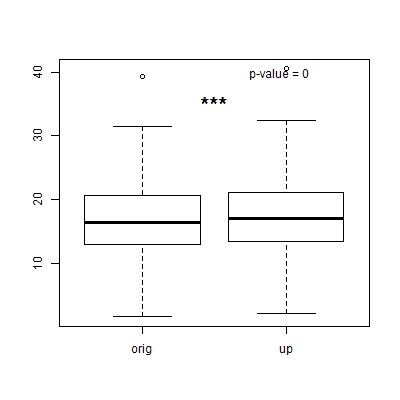

Supplement: S1 File. Data — (ZIP) [file pone.0212280.s001.zip › Evaluation/UpSampleMayo/doc/stat/img/BVTVpairwise.jpg]

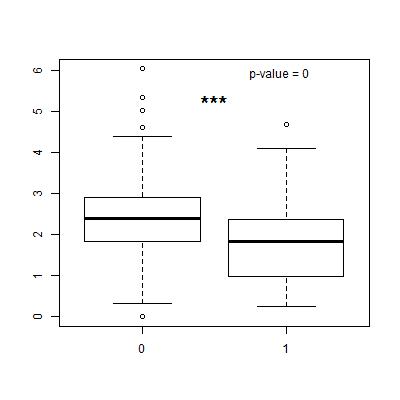

Supplement: S1 File. Data — (ZIP) [file pone.0212280.s001.zip › Evaluation/UpSampleMayo/doc/stat/img/Conn.D_.jpg]

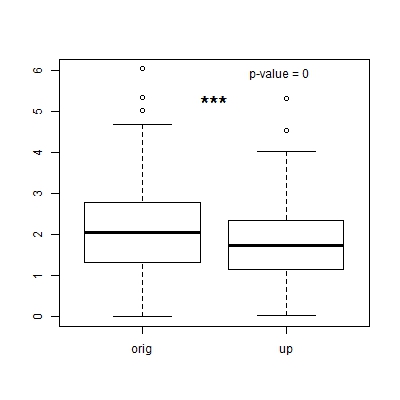

Supplement: S1 File. Data — (ZIP) [file pone.0212280.s001.zip › Evaluation/UpSampleMayo/doc/stat/img/ConnDpairwise.jpg]

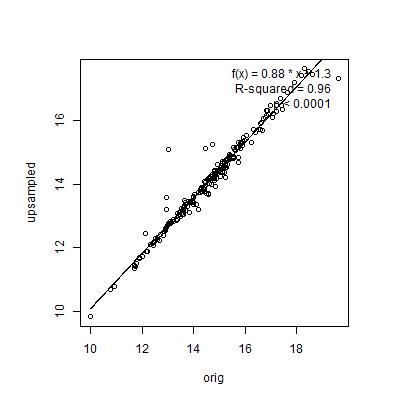

Supplement: S1 File. Data — (ZIP) [file pone.0212280.s001.zip › Evaluation/UpSampleMayo/doc/stat/img/corr_mayo_BSBV.jpg]

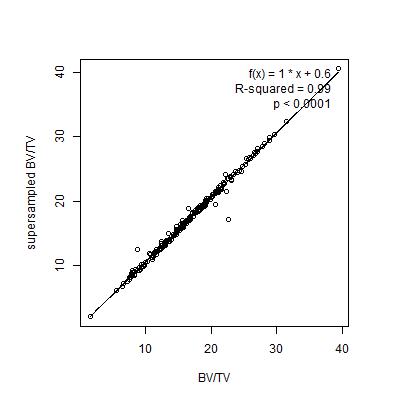

Supplement: S1 File. Data — (ZIP) [file pone.0212280.s001.zip › Evaluation/UpSampleMayo/doc/stat/img/corr_mayo_BVTV.jpg]

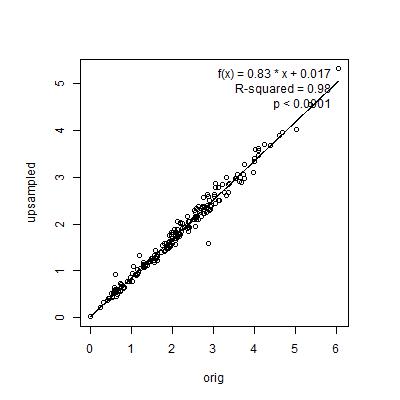

Supplement: S1 File. Data — (ZIP) [file pone.0212280.s001.zip › Evaluation/UpSampleMayo/doc/stat/img/corr_mayo_ConnD.jpg]

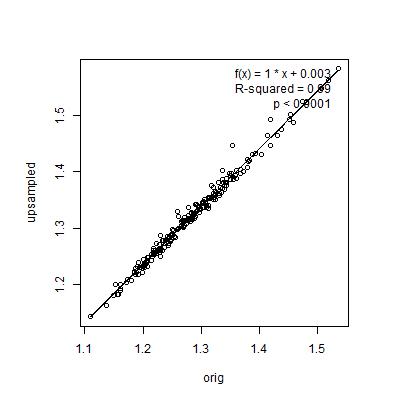

Supplement: S1 File. Data — (ZIP) [file pone.0212280.s001.zip › Evaluation/UpSampleMayo/doc/stat/img/corr_mayo_DA.jpg]

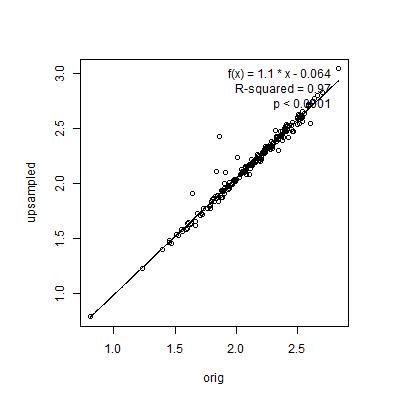

Supplement: S1 File. Data — (ZIP) [file pone.0212280.s001.zip › Evaluation/UpSampleMayo/doc/stat/img/corr_mayo_SMI.jpg]

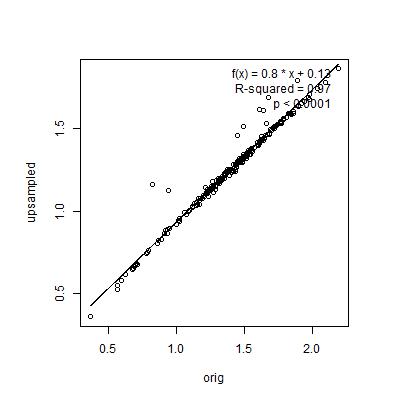

Supplement: S1 File. Data — (ZIP) [file pone.0212280.s001.zip › Evaluation/UpSampleMayo/doc/stat/img/corr_mayo_TbN.jpg]

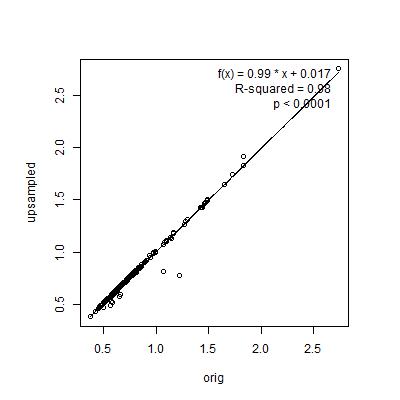

Supplement: S1 File. Data — (ZIP) [file pone.0212280.s001.zip › Evaluation/UpSampleMayo/doc/stat/img/corr_mayo_TbSp.jpg]

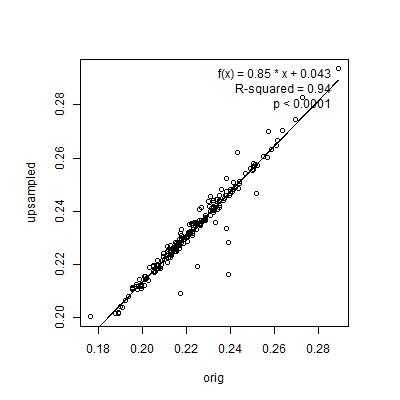

Supplement: S1 File. Data — (ZIP) [file pone.0212280.s001.zip › Evaluation/UpSampleMayo/doc/stat/img/corr_mayo_TbTh.jpg]

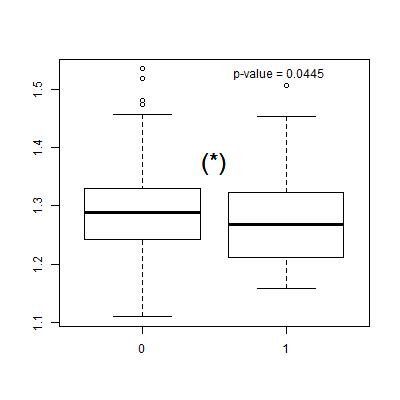

Supplement: S1 File. Data — (ZIP) [file pone.0212280.s001.zip › Evaluation/UpSampleMayo/doc/stat/img/DA_.jpg]

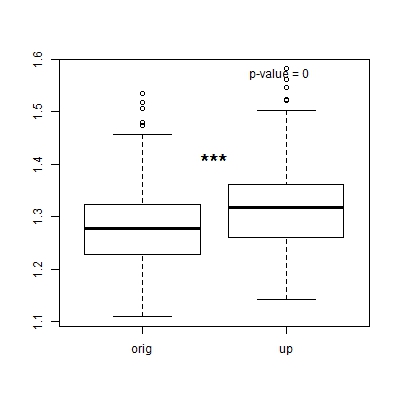

Supplement: S1 File. Data — (ZIP) [file pone.0212280.s001.zip › Evaluation/UpSampleMayo/doc/stat/img/DApairwise.jpg]

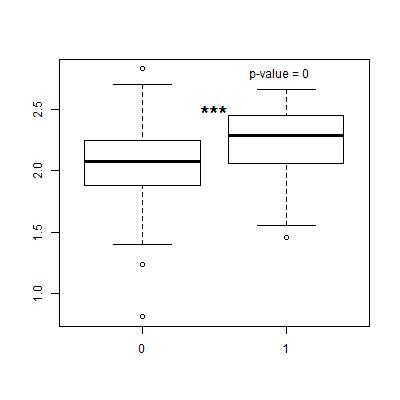

Supplement: S1 File. Data — (ZIP) [file pone.0212280.s001.zip › Evaluation/UpSampleMayo/doc/stat/img/SMI_.jpg]

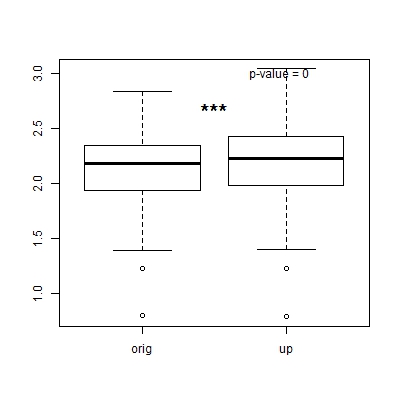

Supplement: S1 File. Data — (ZIP) [file pone.0212280.s001.zip › Evaluation/UpSampleMayo/doc/stat/img/SMIpairwise.jpg]

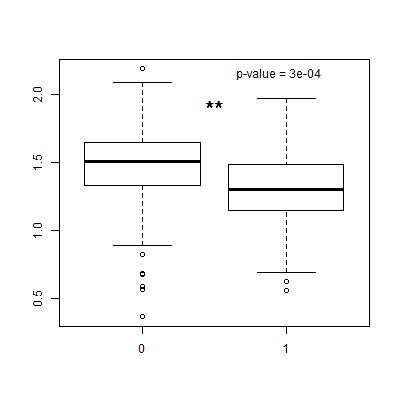

Supplement: S1 File. Data — (ZIP) [file pone.0212280.s001.zip › Evaluation/UpSampleMayo/doc/stat/img/Tb.N_.jpg]

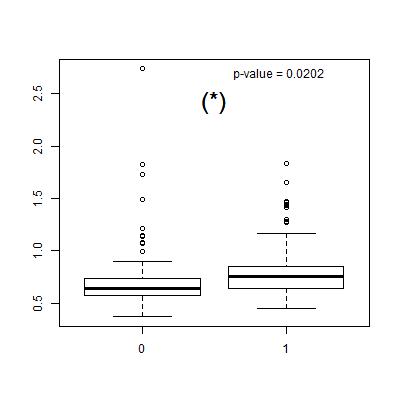

Supplement: S1 File. Data — (ZIP) [file pone.0212280.s001.zip › Evaluation/UpSampleMayo/doc/stat/img/Tb.Sp_.jpg]

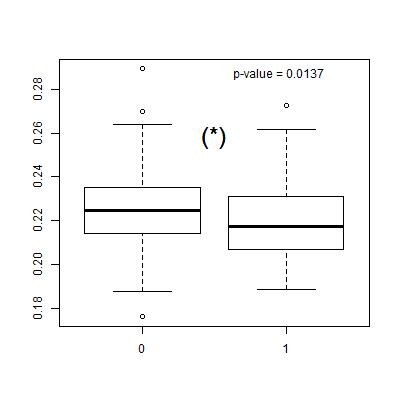

Supplement: S1 File. Data — (ZIP) [file pone.0212280.s001.zip › Evaluation/UpSampleMayo/doc/stat/img/Tb.Th_.jpg]

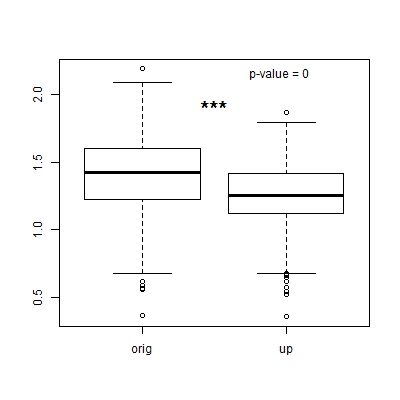

Supplement: S1 File. Data — (ZIP) [file pone.0212280.s001.zip › Evaluation/UpSampleMayo/doc/stat/img/TbNpairwise.jpg]

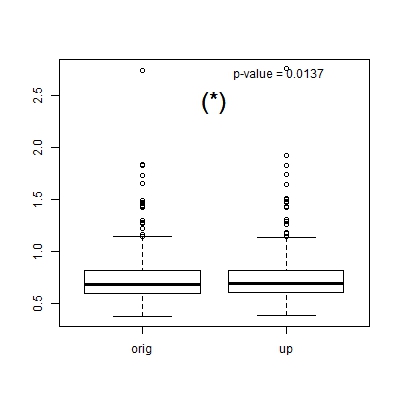

Supplement: S1 File. Data — (ZIP) [file pone.0212280.s001.zip › Evaluation/UpSampleMayo/doc/stat/img/TbSppairwise.jpg]

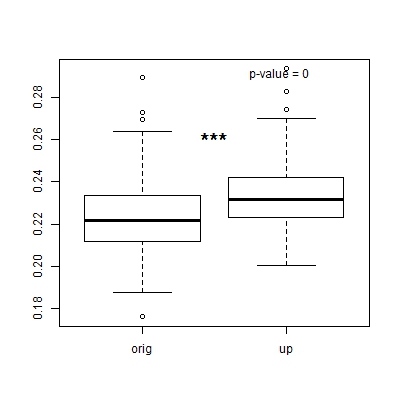

Supplement: S1 File. Data — (ZIP) [file pone.0212280.s001.zip › Evaluation/UpSampleMayo/doc/stat/img/TbThpairwise.jpg]

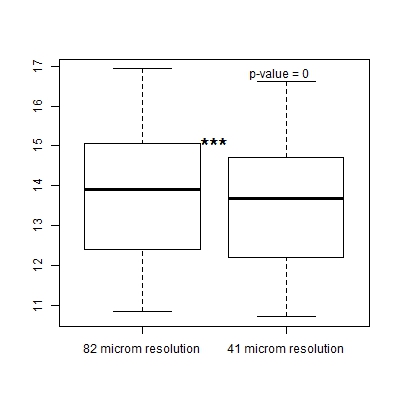

Supplement: S1 File. Data — (ZIP) [file pone.0212280.s001.zip › Evaluation/UpSampleXRepro/doc/stat/img/BSBVpairwise.jpg]

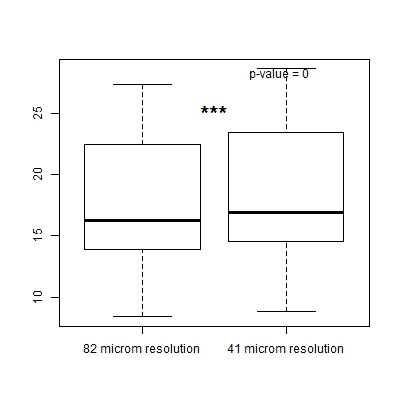

Supplement: S1 File. Data — (ZIP) [file pone.0212280.s001.zip › Evaluation/UpSampleXRepro/doc/stat/img/BVTVpairwise.jpg]

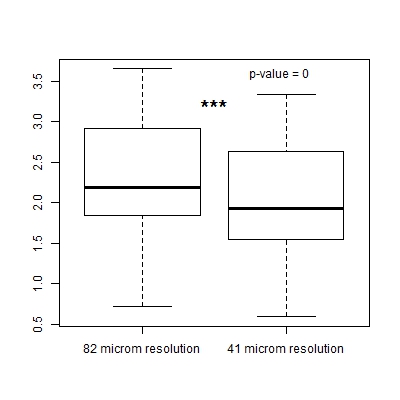

Supplement: S1 File. Data — (ZIP) [file pone.0212280.s001.zip › Evaluation/UpSampleXRepro/doc/stat/img/ConnDpairwise.jpg]

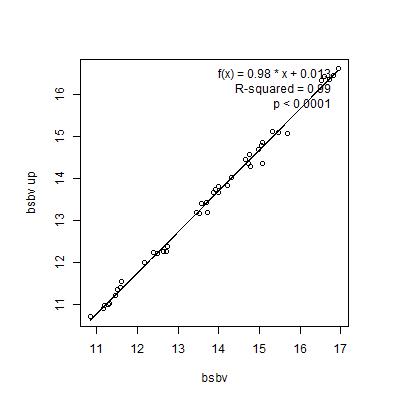

Supplement: S1 File. Data — (ZIP) [file pone.0212280.s001.zip › Evaluation/UpSampleXRepro/doc/stat/img/corr_bsbv.jpg]

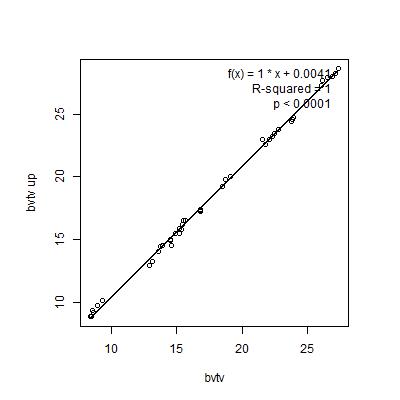

Supplement: S1 File. Data — (ZIP) [file pone.0212280.s001.zip › Evaluation/UpSampleXRepro/doc/stat/img/corr_bvtv.jpg]

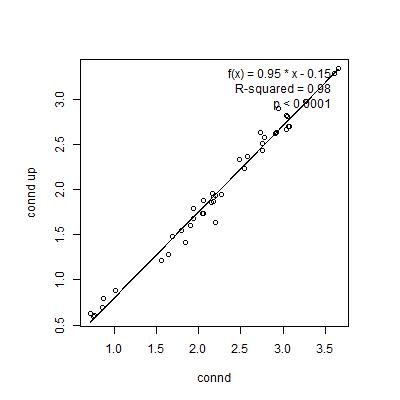

Supplement: S1 File. Data — (ZIP) [file pone.0212280.s001.zip › Evaluation/UpSampleXRepro/doc/stat/img/corr_connd.jpg]

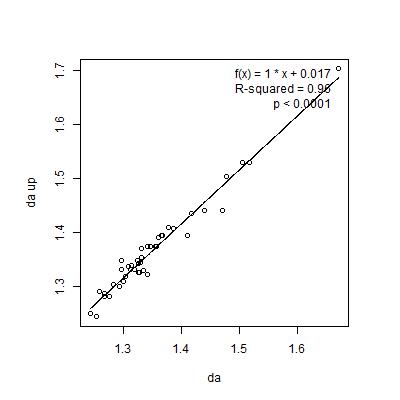

Supplement: S1 File. Data — (ZIP) [file pone.0212280.s001.zip › Evaluation/UpSampleXRepro/doc/stat/img/corr_da.jpg]

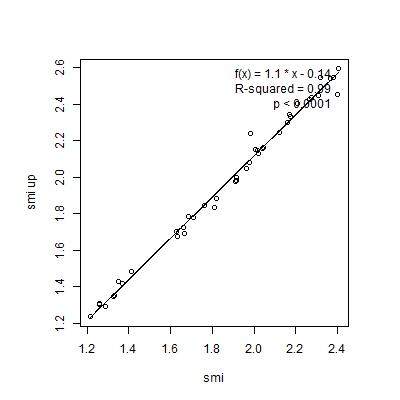

Supplement: S1 File. Data — (ZIP) [file pone.0212280.s001.zip › Evaluation/UpSampleXRepro/doc/stat/img/corr_smi.jpg]

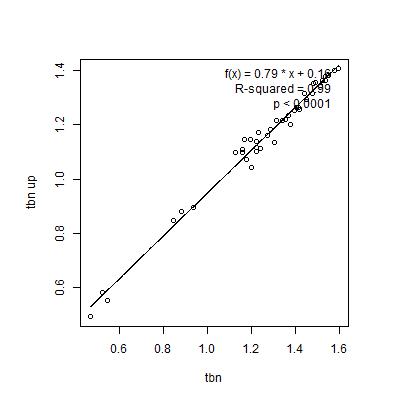

Supplement: S1 File. Data — (ZIP) [file pone.0212280.s001.zip › Evaluation/UpSampleXRepro/doc/stat/img/corr_tbn.jpg]

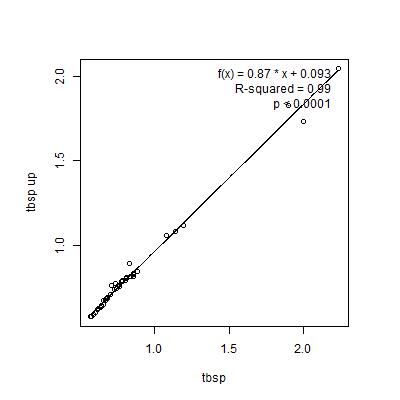

Supplement: S1 File. Data — (ZIP) [file pone.0212280.s001.zip › Evaluation/UpSampleXRepro/doc/stat/img/corr_tbsp.jpg]

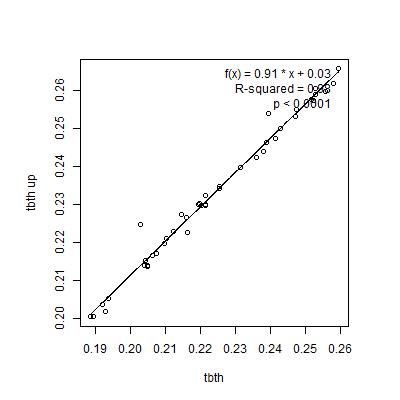

Supplement: S1 File. Data — (ZIP) [file pone.0212280.s001.zip › Evaluation/UpSampleXRepro/doc/stat/img/corr_tbth.jpg]

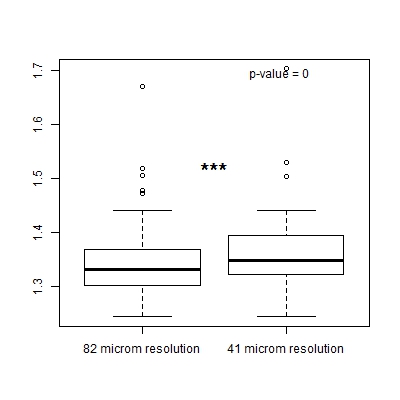

Supplement: S1 File. Data — (ZIP) [file pone.0212280.s001.zip › Evaluation/UpSampleXRepro/doc/stat/img/DApairwise.jpg]

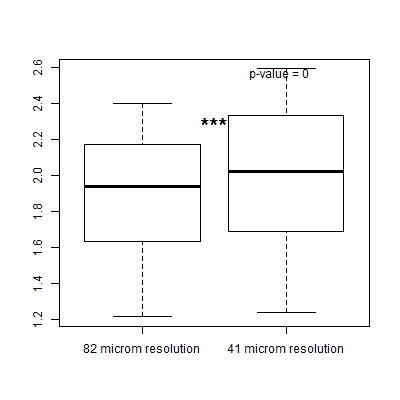

Supplement: S1 File. Data — (ZIP) [file pone.0212280.s001.zip › Evaluation/UpSampleXRepro/doc/stat/img/SMIpairwise.jpg]

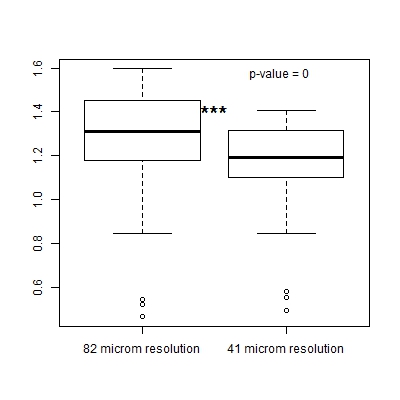

Supplement: S1 File. Data — (ZIP) [file pone.0212280.s001.zip › Evaluation/UpSampleXRepro/doc/stat/img/TbNpairwise.jpg]

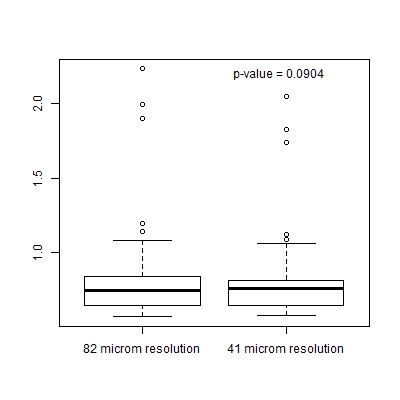

Supplement: S1 File. Data — (ZIP) [file pone.0212280.s001.zip › Evaluation/UpSampleXRepro/doc/stat/img/TbSppairwise.jpg]

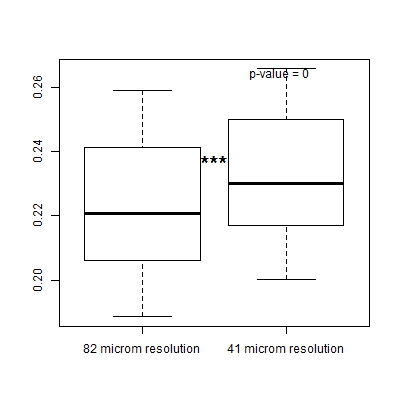

Supplement: S1 File. Data — (ZIP) [file pone.0212280.s001.zip › Evaluation/UpSampleXRepro/doc/stat/img/TbThpairwise.jpg]
